# Decade-Long Trends in Antibiotic Prescriptions According to WHO AWaRe Classification Among Severe Acute Respiratory Infection Patients at Tertiary Hospitals in Bangladesh (2011–2020)

**DOI:** 10.3390/antibiotics14020199

**Published:** 2025-02-14

**Authors:** Fahmida Chowdhury, Saju Bhuiya, Mohammad Abdul Aleem, Tanzir Ahmed Shuvo, Gazi Md. Salahuddin Mamun, Probir Kumar Ghosh, Lubaba Shahrin, Samin Yasar Khan, Md Ariful Islam, Mahmudur Rahman

**Affiliations:** 1 International Centre for Diarrhoeal Disease Research, Bangladesh (icddr,b), Dhaka 1212, Bangladesh; saju.bhuiya@icddrb.org (S.B.); drmdaleem@icddrb.org (M.A.A.); tanzir@icddrb.org (T.A.S.); gazi.mamun@icddrb.org (G.M.S.M.); probir@icddrb.org (P.K.G.); lubabashahrin@icddrb.org (L.S.); arif@icddrb.org (M.A.I.); 2 Centre for Higher Studies and Research (CHSR), Bangladesh University of Professionals (BUP), Dhaka 1216, Bangladesh; 3 Department of Pharmacy and Pharmaceutical Sciences, Monash University, Melbourne, VIC 350108, Australia; skha0083@student.monash.edu; 4 School of Population Health, University of New South Wales (UNSW), Sydney, NSW 2052, Australia; 5 The Eastern Mediterranean Public Health Network (EMPHNET), Dhaka 1212, Bangladesh; mahmudur57@gmail.com

**Keywords:** antibiotic prescriptions, severe acute respiratory infection, hospitals, trends, AWaRe classification

## Abstract

**Background:** To aid in the development of antimicrobial stewardship programs (ASPs), we analyzed the patterns and trends in antibiotic prescriptions for patients with severe acute respiratory infection (SARI), utilizing the WHO’s AWaRe classification. **Methods:** We analyzed data from hospital-based influenza surveillance from January 2011 to December 2020 across nine Bangladeshi tertiary-level hospitals. Surveillance physicians collected WHO-defined SARI patient data, including demographics, clinical characteristics, and antibiotic prescriptions. Descriptive statistics and parametric and non-parametric tests were used for the analysis. **Results:** Of 21,566 SARI patients [median age 20 years (IQR: 1.33–45), 66% male], 91% were prescribed at least one antibiotic. A total of 25,133 antibiotics were prescribed, of which 47.0% were third-generation cephalosporins, 16.5% were macrolides, and 11.1% were beta-lactam/beta-lactamase inhibitors. According to the AWaRe classification, 28.7% were in the Access group, while 71.3% were in the Watch group, and none were from the Reserve group. A downward trend in Access group (30.4% to 25.1%; *p* = 0.010) and an upward trend in Watch group antibiotic prescription (69.6% to 74.9%; *p* = 0.010) were observed. We identified that patients aged < 5 years (aOR: 1.80; 95% CI: 1.44–2.25), who were treated in government hospitals (aOR: 1.45; 95% CI: 1.35–1.57), patients with the presence of lung diseases (aOR: 1.56; 95% CI: 1.35–1.80) had an increased likelihood of being prescribed Watch group antibiotics. **Conclusions**: This study reveals a concerning pattern of antibiotic overuse among SARI patients in Bangladesh, with a growing trend over the past decade towards increased Watch group antibiotic prescriptions. Only one-third of the prescribed antibiotics were from the Access group, falling short of the two-thirds threshold recommended by the WHO. Effective ASPs are crucial to optimize antibiotic prescriptions and mitigate the risk of antimicrobial resistance.

## 1. Introduction

Antimicrobials are crucial for treating infections and have significantly extended the human lifespan [1]. However, the inappropriate prescription of antibiotics poses a serious public health threat, with evidence showing that over half of all prescribed antibiotics are unnecessary [2]. This misuse contributes to antimicrobial resistance (AMR), leading to treatment failures, increased morbidity, mortality, and substantial economic costs [3]. The highest AMR-related death rates are seen in Asia and sub-Saharan Africa, with 124 and 98.9 deaths per 100,000 people, respectively [4]. Globally, AMR directly caused 1.27 million deaths in 2019 and contributed to 4.95 million deaths, and projections suggest up to 10 million deaths annually by 2050 if current trends continue [4,5].

In low- and middle-income countries (LMICs), severe acute respiratory infections (SARIs), caused by various pathogens, pose a major public health challenge [6,7]. Despite the predominantly viral origins of SARIs, antibiotics are frequently prescribed, with inappropriate prescriptions reported in up to 80% of cases [8,9]. This overuse has significantly contributed to AMR, which has led to over 1.5 million deaths from ARIs in 2019 alone [4]. Therefore, the growing crisis of AMR has emerged as a critical global health concern, prompting the World Health Organization (WHO) to declare it a major public health threat [10]. To combat this, the WHO introduced the AWaRe (Access, Watch, Reserve) classification system, recommending that at least 60% of antibiotic use should come from the ‘Access’ group [11,12].

In Bangladesh, AMR is an emerging crisis, with antibiotic consumption in LMICs rising by 76% over the past two decades [13,14]. Surveys indicate that up to 92% of SARI patients received antibiotics during the COVID-19 pandemic, exacerbating the risk of AMR [15,16]. The country has introduced a National Action Plan (NAP) to address AMR, aligned with WHO’s Global Action Plan (GAP) guidelines [17]. However, the effective implementation of the NAP hinges on comprehensive data on AMR and antibiotic prescription trends [13,16]. This study aims to evaluate antibiotic prescription patterns and trends according to the WHO AWaRe classification among severe acute respiratory infection (SARI) patients in tertiary hospitals in Bangladesh, addressing critical data gaps and informing antimicrobial stewardship efforts.

## 2. Results

### 2.1. Demographic and Clinical Characteristics of SARI Patients

Data obtained from a total of 21,566 SARI patients were analyzed. The median age of these patients was 20 years (interquartile range (IQR): 1.33–45 years), with 66% (14,164) being male. The median time from symptom onset to admission was 3 days (IQR: 2–4 days), and the median length of hospital stay was 3 days (IQR: 2–5 days) (Table 1). 

In addition to the primary symptoms of cough and fever—part of the SARI enrolment case definition—the most frequently reported symptoms were difficulty breathing (60.8%, 13,115), headache (56.9%, 8530), and runny nose (47.2%, 10,169) at admission. Among the 6585 children under 5 years, the most frequently reported symptoms were chest indrawing (75.3%, 4958), and inability to drink (24.1%, 1586). Among the 14,981 patients aged ≥ 5 years, 23.1% (3452) had at least one self-reported comorbid condition (Table 1).

Of the SARI patients, 53.1% (11,463) were fully recovered at discharge, while 1.6% (343) died during hospitalization (Table 1).

### 2.2. Antibiotics Prescribed to Treat SARI Patients After Hospital Admission

**Figure 1:**
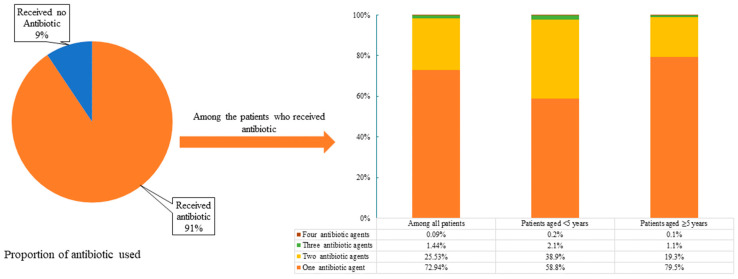
Antibiotics prescribed among SARI patients and number of antibiotic agents prescribed at nine tertiary-level hospitals in Bangladesh (2011–2020).

Among the enrolled SARI patients, 91% (19,531) prescribed a total of 25,133 antibiotics after hospital admission—approximately an average of 1.29 prescriptions per patient. The majority (72.9%, 14,246) of the patients were prescribed one antibiotic agent, while 27.1% (5285) were prescribed ≥ 2 antibiotic agents per patient (Figure 1).

### 2.3. Antibiotics Prescribed to Treat SARI Patients by Age Group

Among the SARI patients aged < 5 years, 93.8% (6178/6585) were prescribed at least one antibiotic, while 41.2% (2544/6178) were prescribed ≥ 2 antibiotic agents. Among the SARI patients aged ≥ 5 years, 89.1% (13,353/14,981) were prescribed at least one antibiotic, while 20.5% (2741/13,353) were prescribed ≥ 2 antibiotics. Moreover, children aged < 5 years had significantly higher rates of antibiotic prescription compared to those aged ≥ 5 years (*p* < 0.001) (Figure 1).

### 2.4. Antibiotics Prescribed to Treat SARI Patients According to Generic Name

During the study period, ceftriaxone was the most frequently prescribed antibiotic (42.9%; 10,783), followed by azithromycin (13.3%; 3335), amoxicillin/clavulanic acid (11.1%; 2793), amikacin (4.1%; 1027), amoxicillin (3.9%; 974), gentamicin (3.7%; 937), cefuroxime (3.7%; 925), flucloxacillin (3.3%; 825), clarithromycin (3.2%; 792), and ceftazidime (1.9%; 474) (Table 2). These ten antibiotics accounted for 90.1% of all antibiotic prescriptions (DU90%).

### 2.5. Trend of Prescribed Antibiotics to Treat SARI Patients According to Generic Name

**Figure 2:**
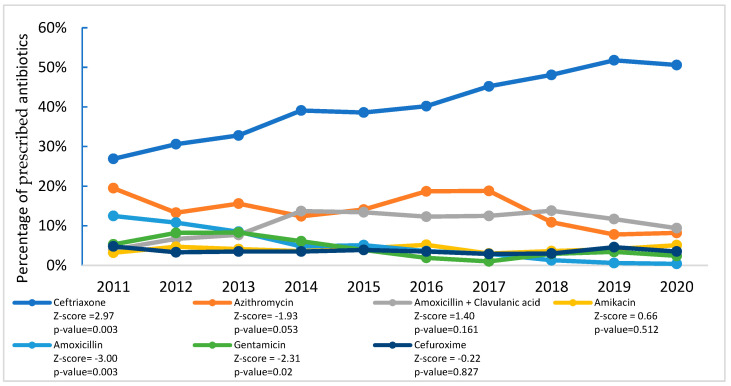
Trends in top seven antibiotics prescribed to treat SARI patients according to generic name of antibiotics at nine tertiary-level hospitals in Bangladesh (2011–2020).

Figure 2 presents temporal shifts in antibiotic frequencies, where a total of 33 antibiotic agents were prescribed to treat SARI patients between 2011 and 2020. Among these, seven antibiotics accounted for 83% of the total antibiotic prescriptions. Ceftriaxone prescription increased significantly from 26.9% in 2011 to a peak of 51.8% in 2019 (*p* = 0.003), before declining slightly to 50.6% in 2020. Conversely, amoxicillin prescriptions decreased significantly from 12.5% in 2011 to 0.4% in 2020 (*p* = 0.003), and gentamicin prescriptions declined from 5.3% to 2.4% (*p* = 0.021). While azithromycin prescription decreased from 19.5% to 8.2% (*p* = 0.053), amoxicillin/clavulanic acid prescription increased from 4.0% to 9.4% (*p* = 0.161), with no significant changes in the trend for amikacin (*p* = 0.512) or cefuroxime (*p* = 0.827) prescription.

### 2.6. Antibiotics Prescribed to Treat SARI Patients According to Antibiotic Classes

**Figure 3:**
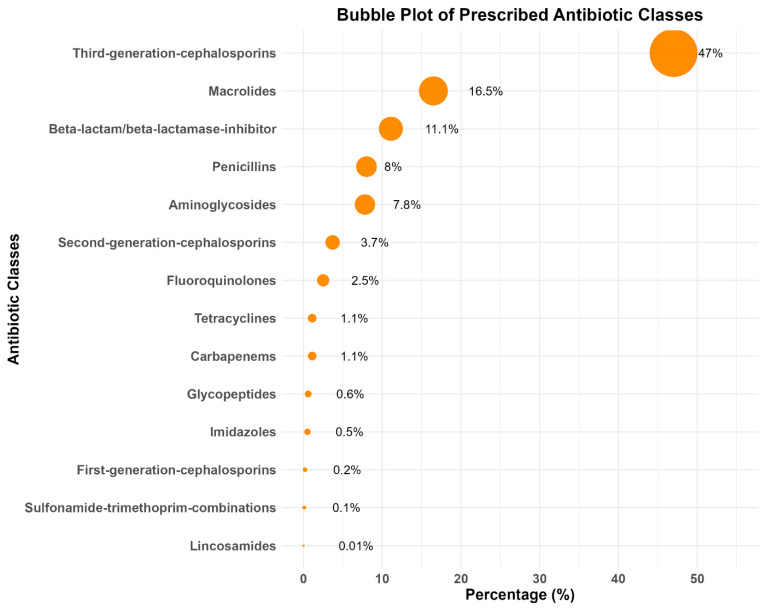
Antibiotics prescribed to treat SARI patients according to antibiotic classes at nine tertiary-level hospitals in Bangladesh (2011–2020).

Among the percentage of prescribed antibiotics, 47.0% (11,806) of the antibiotics were from third-generation cephalosporins, followed by 16.5% (4146) being macrolides, 11.1% (2793) being beta-lactam/beta-lactamase inhibitors, 8.0% (2002) being penicillins, and 7.8% (1966) being aminoglycoside classes of antibiotics (Figure 3).

### 2.7. Trend in Antibiotics Prescribed to Treat SARI Patients According to Different Antibiotic Classes

**Figure 4:**
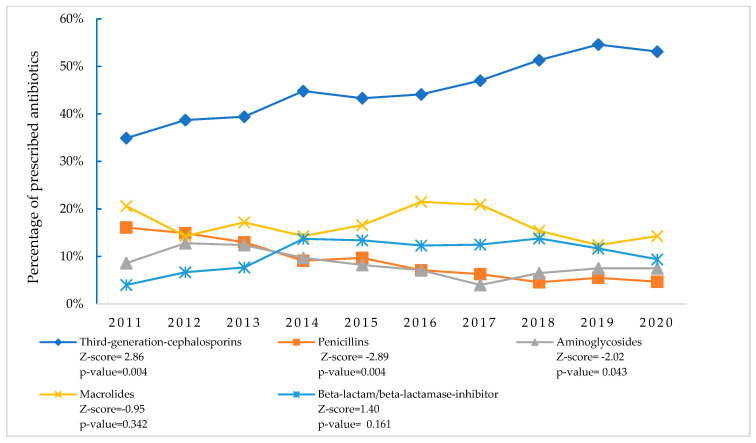
Trends in top five antibiotic classes prescribed to treat SARI patients at nine tertiary-level hospitals in Bangladesh (2011–2020).

During the study period, third-generation cephalosporin prescriptions increased significantly from 34.9% in 2011 to a peak of 54.6% in 2019 (*p* = 0.004). Conversely, penicillin prescriptions decreased significantly from 16.1% to 4.7% (*p* = 0.004), and aminoglycoside prescriptions declined from 8.6% to 7.5% (*p* = 0.043). While macrolide prescriptions decreased from 20.6% to 14.3% (*p* = 0.342), and beta-lactam/beta-lactamase inhibitor prescriptions increased from 4.0% to 9.4% (*p* = 0.161), these changes were not statistically significant (Figure 4).

### 2.8. Antibiotics Prescribed to Treat SARI Patients According to the WHO AWaRe Classification

According to the WHO AWaRe classification, most of the prescribed antibiotics were from the Watch group (71.3%, 17,942), while 28.7% (7191) were from the Access group, and none were from the Reserve group. A total of 33 types of antibiotics were prescribed to treat SARI patients. Of these, 14 antibiotics were from the Access group, and 19 antibiotics were from the Watch group (Table 2).

### 2.9. Antibiotics Prescribed to Treat SARI Patients According to WHO AWaRe Classification by Age Categories

**Figure 5:**
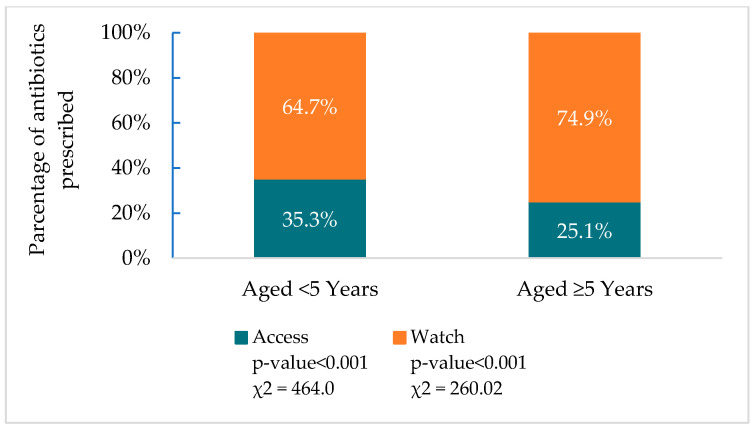
Age-specific antibiotics prescribed according to the WHO AWaRe classification at nine tertiary-level hospitals in Bangladesh (2011–2020).

Of the 8873 antibiotics prescribed to treat 6178 SARI patients aged < 5 years, 64.7% (5742) were from the Watch group, while 35.3% (3131) were from the Access group. A total of 16,260 antibiotics were prescribed to 13,353 patients aged ≥ 5 years; among those antibiotics, 74.9% (12,174) were from the Watch group, and 25.1% (4086) were from the Access group. The prescription of Access group antibiotics (*p* < 0.001) was higher among SARI patients aged < 5 years compared to those aged ≥ 5 years and the prescription of Watch group antibiotics (*p* < 0.001) was higher among SARI patients aged ≥ 5 years compared to those aged < 5 years (Figure 5). A significant difference in the median age was identified between the patients who were prescribed Access group antibiotics and those who were prescribed Watch group antibiotics (14 [0.52–52] vs. 20 [2.5–42]; *p* < 0.001).

### 2.10. Trend of Antibiotic Prescriptions to Treat SARI Patients According to the WHO AWaRe Classification

**Figure 6:**
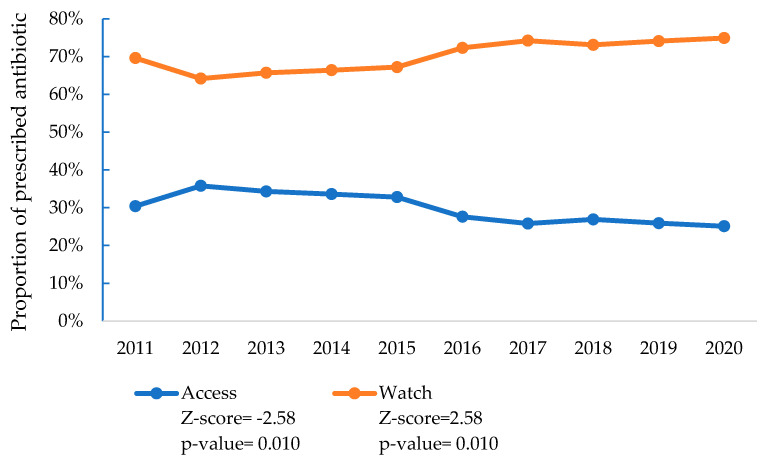
Trends in antibiotics prescribed to treat SARI patients according to WHO AWaRe classification, at nine tertiary-level hospitals in Bangladesh (2011–2020).

Between the years of 2011 and 2012, the Watch group antibiotic prescriptions decreased from 69.6% to 64.2%, while the Access group antibiotic prescriptions increased from 30.4% to 35.8%. From 2012 to 2020, these trends reversed, with Watch group antibiotic prescriptions rising to 74.9% (a sharp increase from 67.2% in 2015 to 72.3% in 2016) and Access group antibiotic prescriptions falling to 25.1% (with a steep decline from 32.8% in 2015 to 27.6% in 2016). No patients were prescribed Reserve group antibiotics during the study period. A significant upward trend in Watch group antibiotic prescriptions (*p* = 0.010) was observed, corresponding to a decrease in Access group antibiotic prescriptions (30.4% to 25.1%; *p* = 0.01) (Figure 6).

### 2.11. Factors Associated with Antibiotic Prescriptions Among SARI Patients

**Figure 7:**
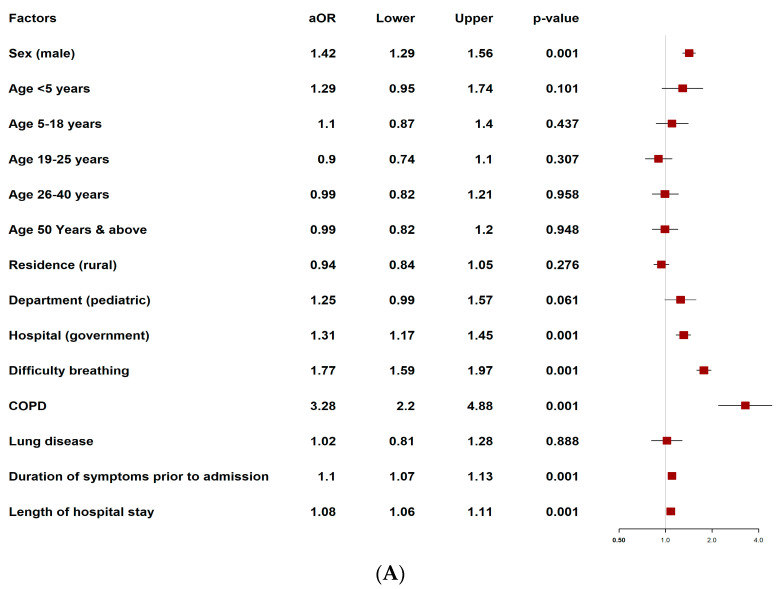

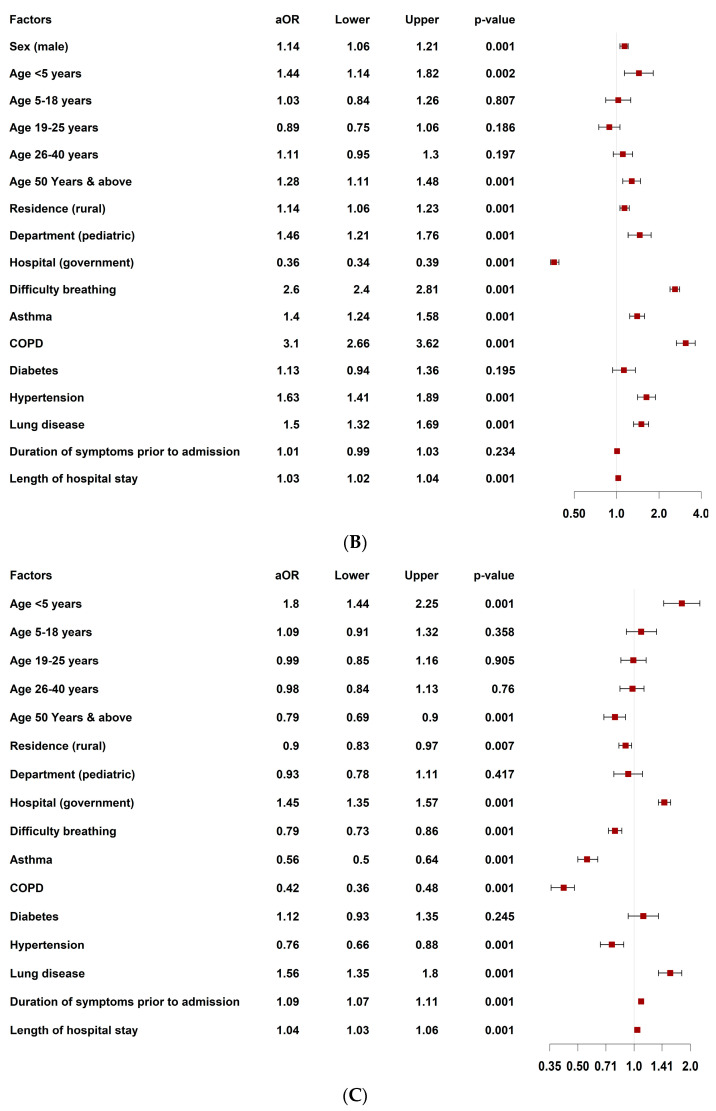
(**A**) Multivariate analysis to identify factors associated with overall antibiotic prescriptions. (**B**) Multivariate analysis to identify factors associated with Access group antibiotic prescriptions. (**C**) Multivariate analysis to identify factors associated with Watch group antibiotic prescriptions. Note: age category: 41–50 years; residence: urban; department: pediatric; hospital: private hospitals were the reference groups. OR: odds ratio, aOR: adjusted odds ratio, COPD: chronic obstructive pulmonary disease.

In the multivariate analysis for overall antibiotic prescriptions, after adjusting for potential confounders, it was observed that the antibiotics were more likely to be prescribed to male patients (adjusted odds ratio (aOR) 1.42, 95% confidence interval (CI) 1.29–1.56) and patients admitted to government hospitals (aOR 1.31, 95% CI 1.17–1.45) (Figure 7A, Table S1(A)). Additionally, patients with COPD (aOR 3.28; 95% CI 2.20–4.88), difficulty breathing (aOR 1.77, 95% CI 1.59–1.97), a longer duration of symptoms prior to admission (aOR 1.10; 95% CI: 1.07–1.13), and a longer duration of hospital stay (aOR 1.08; 95% CI 1.06–1.11) were more likely to be prescribed antibiotics (Figure 7A, Table S1(A)).

A separate multivariate analysis revealed several factors associated with Access group antibiotic prescriptions. These prescriptions were more likely in male patients (aOR 1.14; 95% CI 1.06–1.21), children under 5 years (aOR 1.44; 95% CI 1.14–1.82), elderly patients aged ≥ 50 years (aOR 1.28; 95% CI 1.11–1.48), patients living in rural areas (aOR 1.14; 95% CI 1.06–1.23), and patients admitted to pediatric departments (aOR 1.46, 95% CI 1.21–1.76) (Figure 7(B), Table S1(B)). Additionally, patients with COPD (aOR 3.10, 95% CI 2.66–3.62), difficulty breathing (aOR 2.60; 95% CI 2.40–2.81), hypertension (aOR 1.63; 95% CI 1.41–1.89), lung diseases (aOR 1.50; 95% CI 1.32–1.69), and asthma (aOR 1.40; 95% CI 1.24–1.58) were more likely to be prescribed Access group antibiotics. The longer duration of hospital stay was also positively associated with Access group antibiotic prescriptions (aOR 1.03; 95% CI 1.02–1.04) (Figure 7B, Table S1(B)).

In another multivariate model after adjusted confounders, we revealed that Watch group antibiotics were more likely prescribed to children under 5 years (aOR 1.80; 95% CI 1.44–2.25), and patients admitted to government hospitals (aOR 1.45; 95% CI 1.35–1.57) (Figure 7C, Table S1(C)). Additionally, patients with lung diseases (aOR 1.56; 95% CI: 1.35–1.80), a longer duration of symptoms prior to admission (aOR 1.09; 95% CI: 1.07–1.11), and a longer duration of hospital stay (aOR 1.04; 95% CI: 1.03–1.06) were associated with an increased likelihood of Watch group antibiotic prescription (Figure 7C, Table S1(C)).

## 3. Discussion

Our study revealed concerning antibiotic prescription patterns among SARI patients, with 91% of the patients prescribed antibiotics during hospitalization. This rate is notably higher than that in previous reports in Bangladesh (78%) and China (85.6%) [16,18]. In contrast, lower rates have been observed in the USA (41%) and India (45.1%) [19,20]. Such high prescription rates, particularly for SARI, which can mimic bacterial infections, may lead to unnecessary antibiotic prescriptions.

Additionally, 27% of the patients were prescribed multiple antibiotics, which is lower than the 37.8% observed in China [18]. The higher rate of antibiotic prescriptions in younger children (<5 years) compared to older patients may reflect perceptions of increased susceptibility or the presence of resistant pathogens in hospital settings. This discrepancy warrants further investigation into factors influencing prescribing practices for young children [16,21]. Moreover, pharmaceutical marketing in Bangladesh contributes to the misuse of antibiotics. Aggressive promotion by companies, coupled with wide availability, often leads to physicians overprescribing antibiotics for minor illnesses, exacerbating the issue of AMR. This study underscores the critical need for effective antibiotic stewardship and more stringent controls on antibiotic prescriptions to prevent the escalation of resistance.

Our study highlights a predominant prescription of third-generation cephalosporins, macrolides, beta-lactam/beta-lactamase inhibitors, and penicillins. This is consistent with findings from a point prevalence survey in Bangladesh, a meta-analysis of antibiotic use in Iran, multicentric studies in India and Nigeria, and national trend analyses in US hospitals [16,22,23,24,25]. The significant use of third-generation cephalosporins surpasses the rates reported in India (24.5%) and globally (24.2%) [23,26]. This trend is concerning as it contributes to the emergence of extended-spectrum beta-lactamase (ESBL)-producing pathogens, which are highly resistant bacteria [27,28]. Macrolides were the second most commonly prescribed antibiotic class (16.5%) in our study. While not typically among the top five prescribed antibiotics in many countries, including India, their overuse can also raise concerns [23,26]. Additionally, the overprescription of macrolides raises concerns about the reduced susceptibility of pathogens such as Streptococcus pneumoniae and Salmonella typhi [26,29,30,31], potentially jeopardizing the effectiveness of the antimicrobial stewardship program (ASP) in Bangladesh.

A key finding is that 71.3% of the prescribed antibiotics were from the WHO’s AWaRe Watch group, indicating a significant number of antibiotics prescribed with a higher resistance potential. Importantly, no Reserve group antibiotics were prescribed. A recent study on Bangladesh’s antimicrobial supply chain found that 64% of the top-selling antibiotics were from the Watch group, which is consistent with our results [32]. Similarly, 57.9% of the antibiotics consumed in India and 56.6% in China are from the Watch group [18,23]. In contrast, only 5.6% of the ARI patients in Vietnam received Watch group antibiotics, due to national restrictions on their reimbursement [33]. This evidence supports the development of an ASP to reduce the prescription of Watch group antibiotics and increase the appropriate prescription of Access group antibiotics.

The WHO recommends that at least 60% of the national antibiotic consumption should come from the Access group [34]. However, our study found the complete opposite in Bangladesh, which showed that only 28.7% of the consumption was of Access group antibiotics, with 71.3% of the consumption being of Watch group antibiotics. The data from our study represent a specific subset of patients with respiratory infections, which may not be representative of the broader population and prescribing practices across the country. Nationwide antibiotic consumption would require data from a wider range of healthcare settings, conditions, and patient groups. Therefore, while this result highlights prescribing patterns within the study group, it does not provide an accurate measure of the national antibiotic consumption. This discrepancy underscores the need for further research and analysis to accurately assess national antibiotic consumption patterns and identify strategies to optimize antibiotic prescriptions in Bangladesh. Watch group antibiotics, being mostly broad-spectrum, heighten the risk of AMR by allowing resistant strains to proliferate, potentially leading to difficult-to-treat infections [34]. Additionally, broad-spectrum antibiotics disrupt the body’s natural bacterial balance, increasing the risk of superinfections with resistant strains [35,36]. These results highlight the urgent need for better antibiotic stewardship. Despite national action plans to tackle AMR in the WHO South-East Asia Region, effective implementation, especially in Bangladesh, remains challenging [37].

Our study noted an initial increase in Access group antibiotic (narrow-spectrum) prescriptions from 2011 to 2012, but a concerning trend emerged with rising ceftriaxone (a broad-spectrum Watch group antibiotic) prescriptions over the next eight years. In contrast, amoxicillin (an Access group antibiotic) prescription declined despite WHO prioritizing its use. The dominance of ceftriaxone raises concerns about overprescription and AMR. While azithromycin, amoxicillin/clavulanic acid, amikacin, and cefuroxime showed no significant trends, azithromycin and cefuroxime prescriptions decreased notably. Statistically significant declines in penicillins and aminoglycosides suggest growing bacterial resistance to these classes.

Our study highlights a troubling trend in Bangladeshi tertiary hospitals: a growing reliance on Watch group antibiotic prescriptions for SARI patients, coupled with a decline in the prescription of Access group antibiotics. Addressing this issue requires promoting the appropriate prescription of narrow-spectrum antibiotics and implementing an effective ASP. The absence of Reserve group antibiotic prescriptions throughout the study period is a positive finding. Gaining insight into physicians’ understanding of the WHO AWaRe classification and their prescribing practices could be valuable. Additionally, further research on the impact of antibiotic stewardship programs in Bangladeshi hospitals is needed.

Our study has revealed several factors influencing overall antibiotic prescriptions among hospitalized SARI patients. Males were more likely to be prescribed antibiotics than females, particularly Access group antibiotics, a finding consistent with studies from Bangladesh and China, although these studies did not specifically analyze antibiotic use according to the AWaRe classification [18,20,38]. This may reflect variations in symptom presentation or underlying health conditions more prevalent in males, leading to a preference for Access, narrower-spectrum antibiotics and a reduced risk of resistance development. Watch group antibiotic prescriptions were more common in government hospitals compared to private facilities, mirroring a trend observed in a Bangladesh study on young children with ARIs [38]. Resource constraints and high patient volumes in government hospitals may contribute to the more frequent precautionary use of broad-spectrum Watch group antibiotics, despite the risk of increasing antimicrobial resistance [39]. Comorbidities such as COPD and difficulty breathing significantly increased Access group antibiotic prescriptions, aligning with findings from Ethiopia and the United States [20,40]. However, these studies also did not specifically analyze antibiotic use based on the AWaRe classification. This finding highlights the reliance on these antibiotics in managing severe respiratory conditions, where timely and effective treatment is critical. Access group antibiotics might have been preferred in these scenarios to avoid the overuse of broad-spectrum antibiotics. Although some studies report minimal association between antibiotic use and preexisting comorbidities [18,41], our study found that prolonged symptom duration before admission and longer hospital stays were linked to increased Watch group antibiotic prescriptions, consistent with ARI studies in United States and COVID-19 research [20,42]. Patients with lingering symptoms or those requiring longer hospitalization are likely perceived as having more complex or advanced infections, prompting the use of broad-spectrum antibiotics as a means to cover a wide range of potential pathogens.

Our findings highlight the urgent need for effective antibiotic stewardship programs nationwide. The WHO AWaRe classification provides a valuable framework for promoting appropriate antibiotic prescriptions. England’s adaptation of the WHO Essential Medicines List has led to increased Access group antibiotic prescriptions, demonstrating the benefits of tailoring stewardship practices to local needs [43]. Effective antimicrobial stewardship requires implementing national guidelines, training healthcare workers, and conducting regular audits [44]. Similar approaches, adapted to our specific context, could significantly improve antibiotic prescriptions in Bangladesh. For instance, programs in England and sub-Saharan Africa have shown positive results by aligning local policies with WHO recommendations [43,45]. Moreover, utilizing rapid diagnostic tests (RDTs) could be effective in reducing irrational antibiotic prescriptions in resource-limited settings such as Bangladesh [5].

This study benefits from a large sample size, allowing for a robust statistical analysis. However, such a sample size may detect statistically significant differences that might not be clinically relevant. The focus on tertiary hospitals limits the generalizability, as their patient population may not represent the broader healthcare system in Bangladesh. This study’s geographical limitations include unrepresented regions in north–central and southeastern Bangladesh, an urban bias in selected sites, the underrepresentation of rural populations, and overlooked disparities in healthcare access and environmental factors. These factors may affect the generalizability of findings, highlighting the need for expanded regional coverage in future studies. Additional limitations include the lack of data on disease severity, detailed clinical contexts, patient demographics, antibiotic preferences, premature discharge, and external pressures influencing prescribing patterns. Furthermore, the data reflect antibiotic prescriptions only at enrollment, within 24 h of hospital admission, and do not account for changes in antibiotic prescriptions during longer hospital stays.

## 4. Materials

### 4.1. Study Settings and Population

**Figure 8:**
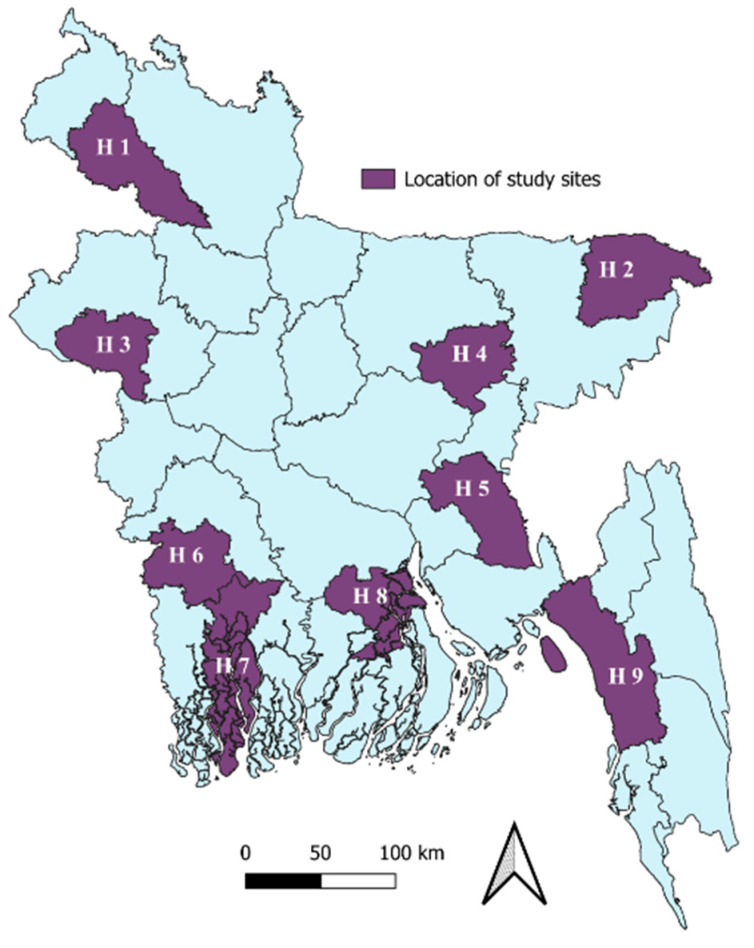
Geographic distribution of nine study sites across Bangladesh.

This observational study analyzed antibiotic use utilizing secondary data from hospital-based influenza surveillance (HBIS) in Bangladesh from January 2011 to December 2020. The HBIS was implemented by the International Centre for Diarrhoeal Disease Research, Bangladesh (icddr,b), and the Institute of Epidemiology, Disease Control and Research (IEDCR) of the Government of Bangladesh (GoB). Through HBIS, patients with SARI were identified from nine tertiary-level hospitals across different geographical locations of Bangladesh (Figure 8). Detailed surveillance methods have been published elsewhere [46].

### 4.2. Inclusion Criteria, Case Identification, and Data Collection 

Surveillance physicians prospectively monitored hospital admissions for individuals who met the WHO-defined SARI case definition (hospitalization with a history or measured fever ≥38 °C, cough, and symptom onset within the past 10 days), which varied slightly over the study period, as described elsewhere [46]. The patients who met the SARI case definition in the selected departments of nine hospitals were considered as eligible for the study meeting the inclusion criteria.

After identifying SARI patients and obtaining written informed consent, patients were enrolled in the study. Data on demographics, clinical characteristics, age, sex, duration of illness, medical history, history of antibiotics received after hospitalization, comorbid conditions, and preliminary diagnosis were collected.

### 4.3. WHO’s AWaRe Classification of Antibiotics

In this study, prescribed antibiotics were categorized according to the AWaRe (Access, Watch, Reserve) classification developed by the WHO in 2017 and updated in 2019. This classification system aids in the development of tools for antibiotic stewardship, reduces antimicrobial resistance, and underscores the importance of appropriate antibiotic use [47].

### 4.4. Statistical Analysis

Descriptive statistics analyzed antibiotic use data categorized by antibiotic types, generic names and AWaRe classes. The most commonly used antibiotic patterns were analyzed using the DU90% index [48]. The drug utilization 90% (DU90%) index was calculated to identify the number of drugs contributing to 90% of the total drug use. The Chi-square (χ²) test was employed to examine associations between categorical variables. Temporal trends in antibiotic prescriptions were evaluated using Cochran–Armitage trend analysis, with statistical significance set at *p*-value ≤ 0.05 [49,50]. To identify factors associated with overall antibiotic prescription, including Access and Watch group antibiotics, univariate and multivariate logistic regression analyses were performed to estimate adjusted odds ratios (aORs) with 95% confidence intervals (CIs) with statistical significance set at *p*-value ≤ 0.05. These multivariable model analyses controlled for potential confounders and covariates, including age, sex, residence, department, hospital, location, and specific health conditions. Every SARI admission was considered as an independent event. All analyses were performed using Stata 15.0 software (StataCorp. 2017) and R software (version 4.3.1) within the RStudio environment (version 2023.06.1+524, RStudio, Inc., Boston, MA, USA).

## 5. Conclusions

This decade-long study reveals a concerning trend in antibiotic overuse in Bangladeshi tertiary hospitals among SARI patients, characterized by a high volume of antibiotic prescriptions and frequent reliance on broad-spectrum, Watch group antibiotics. The antibiotic use was particularly high among children under five, highlighting the need for targeted training on rational antibiotic prescribing for pediatricians. The increasing proportion of Watch group antibiotic prescriptions and coupled with a decline in Access group antibiotic prescriptions suggest rising AMR risks. This study highlights the need for effective ASPs in Bangladeshi hospitals to promote the responsible prescription of antibiotics. This ASP should ensure patients were prescribed proper medication at the correct dose for the right duration. Moreover, promoting the use of narrower-spectrum, Access group antibiotics, as per WHO AWaRe recommendations, is essential. Additionally, interventions such as point-of-care testing and education on treating respiratory viral infections can help reduce unnecessary antibiotic use. Implementing robust antimicrobial stewardship programs with training on evidence-based antibiotic use and rational prescribing practices is critical. These measures could significantly curb irrational antibiotic use and AMR in Bangladesh, providing valuable insights for ASP development in LMICs.

## Figures and Tables

**Table 1:** Patient demographics, clinical characteristics, and outcomes of SARI patients at nine tertiary-level hospitals in Bangladesh (2011–2020).

Characteristics	SARI Patients
	N = 21,566
	n (%)
**Age (in years)**	
0–5	6585 (30.5)
6–14	2621 (12.2)
15–49	7450 (34.6)
50–59	1667 (7.7)
≥60	3243 (15.0)
Median (IQR)	20 (1.33–45)
**Sex**	
Male	14,164 (65.7)
Duration of symptoms prior to admission in days; median (IQR)	3 (2–4)
Length of hospital stay in days; median (IQR)	3 (2–5)
**Symptoms at enrollment of all SARI cases along with fever and cough**	
Difficulty breathing	13,115 (60.8)
Runny nose	10,169 (47.2)
Headache (aged ≥ 5 years; n = 14,980)	8530 (56.9)
Sore throat	2798 (13.0)
**Symptoms at enrollment of SARI (Patients aged < 5 years; N = 6585)**	
Chest indrawing	4958 (75.3)
Unable to drink	1586 (24.1)
Vomiting	869 (13.2)
Lethargy	212 (3.2)
Stridor	187 (2.8)
**Comorbid condition (Self-reported) (Patients aged ≥ 5 years; N = 14,981)**	
≥1 preexisting comorbid condition	3452 (23.1)
Asthma	1442 (6.7)
Lung disease	1382 (6.4)
Hypertension	1101 (5.1)
COPD	1032 (4.8)
Diabetes	684 (3.2)
Heart diseases	169 (0.8)
Kidney disease	58 (0.3)
Liver disease	23 (0.1)
Cancer	17 (0.1)
**Chest X-ray findings (who had chest radiograph; N = 9589)**	
Normal	4694 (49.0)
Lobar consolidation	1378 (14.4)
Pleural effusion	473 (4.9)
Hyperinflation	414 (4.3)
Patchy opacities	224 (2.3)
Alveolar infiltrate	116 (1.2)
Pneumothorax	77 (0.8)
Interstitial infiltrate	73 (0.8)
Other	2140 (22)
**Physician’s diagnosis at enrollment**	
**Aged < 5 years** (n = 6585)	
Pneumonia	4500 (68.3)
Bronchiolitis	762 (11.6)
Acute Respiratory Infection	519 (7.9)
Viral fever	154 (2.3)
Bronchial asthma	41 (0.6)
Bronchitis	28 (0.4)
Others	581 (8.8)
** Aged ≥ 5 years (n = 14,981)**	
Acute respiratory infection	4093 (27.4)
Viral fever	2373 (15.8)
COPD	1726 (11.5)
Bronchial asthma	1379 (9.2)
Fever under evaluation	1226 (8.2)
Pneumonia	1176 (7.8)
Acute febrile illness	761 (5.1)
Enteric fever	327 (2.2)
Suspected COVID-19	136 (0.9)
Dengue	87 (0.6)
Bronchiolitis	52 (0.3)
Others	1645 (11)
**Clinical outcome**	
Fully recovered	11,463 (53.1)
Partially recovered	9380 (43.5)
Transferred to specialized hospitals	380 (1.8)
Death	343 (1.6)

Abbreviations: SARI: severe acute respiratory infections; IQR: interquartile range; COPD: chronic obstructive pulmonary disease.

**Table 2:** Antibiotics prescribed (according to generic name) to treat SARI patients at nine tertiary-level hospitals in Bangladesh (2011–2020).

Sl.	Name of Antibiotic	AWaRe Classification	Total Antibiotics Prescribed N = 25,133 n (%)
Drug Utilization 90%, 1–10			22,870 (91%)
1	Ceftriaxone	Watch	10,783 (42.9)
2	Azithromycin	Watch	3338 (13.3)
3	Amoxicillin/clavulanic acid	Access	2793 (11.1)
4	Amikacin	Access	1028 (4.1)
5	Amoxicillin	Access	974 (3.9)
6	Gentamicin	Access	937 (3.7)
7	Cefuroxime	Watch	925 (3.7)
8	Flucloxacillin	Access	825 (3.3)
9	Clarithromycin	Watch	792 (3.2)
10	Ceftazidime	Watch	475 (1.9)
**Others 11–33**			**2277 (9)**
11	Levofloxacin	Watch	403 (1.6)
12	Cefixime	Watch	333 (1.3)
13	Meropenem	Watch	271 (1.1)
14	Doxycycline	Access	257 (1.0)
15	Ciprofloxacin	Watch	210 (0.8)
16	Cefotaxime	Watch	188 (0.7)
17	Vancomycin	Watch	145 (0.6)
18	Metronidazole	Access	128 (0.5)
19	Ampicillin	Access	88 (0.3)
20	Cloxacillin	Access	62 (0.2)
21	Cephradine	Access	38 (0.1)
22	Benzathine penicillin	Access	29 (0.1)
23	Cefpodoxime	Watch	27 (0.1)
24	Phenoxymethyl penicillin	Access	20 (0.1)
25	Erythromycin	Watch	16 (0.06)
26	Tetracycline	Watch	14 (0.04)
27	Cotrimoxazole	Watch	12 (0.05)
28	Cephalexin	Watch	5 (0.02)
29	Gatifloxacin	Watch	5 (0.02)
30	Pivmecillinam	Watch	4 (0.02)
31	Cefaclor	Access	4 (0.02)
32	Clindamycin	Access	3 (0.01)
33	Streptomycin	Watch	1 (0.00)

## Data Availability

According to data policies of the contributing institutions, to protect intellectual property rights, the primary data cannot be made publicly available by the authors. The data may be made available upon reasonable request to the Institutional Data Access Committees (IDAC) of the contributing institutions.

## Supplementary Materials

The following supporting information can be downloaded at https://www.mdpi.com/article/10.3390/antibiotics14020199/s1: Table S1: Univariate logistic regression model identifying factors associated with antibiotic prescriptions to treat SARI patients at nine tertiary hospitals in Bangladesh (2011–2020). (A) Factors associated with overall antibiotic prescriptions. (B) Factors associated with Access group antibiotic prescription. (C) Factors associated with Watch group antibiotic prescriptions.
