# Supplementary material for: Decade-Long Trends in Antibiotic Prescriptions According to WHO AWaRe Classification Among Severe Acute Respiratory Infection Patients at Tertiary Hospitals in Bangladesh (2011–2020)
Source: Antibiotics (Basel). 2025 Feb 14;14(2):199. doi: 10.3390/antibiotics14020199 (PMC11852029; doi:10.3390/antibiotics14020199)
Supplement: Supplementary file 1 [file antibiotics-14-00199-s001.zip › antibiotics-3382678-supplementary-done.pdf]

antibiotics

MDPI

Supplementary Materials:

Table S1: Univariate logistic regression model identifying factors associated with antibiotic prescriptions to treat SARI patients at nine tertiary hospitals in Bangladesh (2011–2020). **(A)** Factors associated with overall antibiotic prescriptions. **(B)** Factors associated with Access group antibiotic prescription. **(C)** Factors associated with Watch group antibiotic prescriptions.

**A.**				
**A. Overall antibiotic prescribed**	**Univariate**			
**Factors**	**OR**	**lower**	**upper**	**p-value**
Sex (male)	1.24	1.17	1.32	<0.001
<5 years	1.90	1.58	2.29	<0.001
5-18 years	1.10	0.91	1.34	0.313
19-25 Years	0.74	0.61	0.90	0.003
26-40 Years	0.91	0.75	1.10	0.308
50 Years & above	1.30	1.08	1.57	0.006
Residence (rural)	1.54	1.43	1.65	<0.001
Department (pediatric)	1.68	1.53	1.86	<0.001
Hospital (government)	1.47	1.33	1.63	<0.001
Difficulty breathing	2.21	2.01	2.42	<0.001
Asthma	1.06	0.88	1.27	0.571
COPD	3.88	2.66	5.67	<0.001
Diabetes	1.11	0.84	1.45	0.461
Hypertension	0.97	0.79	1.19	0.742
Kidney diseases	1.10	0.44	2.77	0.832
Heart diseases	0.77	0.48	1.24	0.286
Lung disease	1.49	1.20	1.85	<0.001
Duration of symptoms prior to admission	1.08	1.05	1.11	<0.001
Length of hospital stay	1.04	1.11	1.16	<0.001

**B**				
**A. Access group antibiotic prescribed**	**Univariate model**			
**Factors**	**OR**	**lower**	**upper**	**p-value**
Sex (male)	1.24	1.17	1.32	<0.001
<5 years	2.27	2.00	2.58	<0.001
5-18 years	0.97	0.84	1.11	0.626
19-24 Years	0.57	0.49	0.68	<0.001
25-40 Years	0.85	0.73	0.98	0.03
50 Years & above	2.02	1.77	2.30	<0.001
Residence (rural)	1.54	1.44	1.65	<0.001

Department (pediatric)	1.66	1.57	1.76	<0.001
Hospital (government)	0.44	0.41	0.47	<0.001
Difficulty breathing	3.16	2.96	3.38	<0.001
Asthma	1.42	1.28	1.59	<0.001
COPD	4.54	3.98	5.17	<0.001
Diabetes	1.36	1.16	1.59	<0.001
Hypertension	1.94	1.72	2.2	<0.001
Kidney diseases	1.02	0.58	1.78	0.944
Heart diseases	1.22	0.89	1.67	0.224
Lung disease	2.52	2.26	2.82	<0.001
Duration of symptoms prior to admission	0.96	0.95	0.98	<0.001
Length of hospital stay	1.05	1.04	1.06	<0.001

C

**C. Watch group antibiotic prescription**	**Univariate model**			
**Factors**	**OR**	**lower**	**upper**	**p-value**
Sex (male)	1.04	0.98	1.12	0.203
Aged <5 years	1.70	1.49	1.95	<0.001
Aged 5-18 years	1.18	1.02	1.36	0.022
Aged 19-24 Years	1.12	0.96	1.31	0.144
Aged 25-40 Years	1.07	0.93	1.24	0.353
Aged 50 Years & above	0.66	0.58	0.75	<0.001
Residence (rural)	0.82	0.76	0.88	<0.001
Department (pediatric)	1.73	1.61	1.85	<0.001
Hospital (government)	1.47	1.37	1.58	<0.001
Difficulty breathing	0.82	0.77	0.88	<0.001
Asthma	0.48	0.43	0.53	<0.001
COPD	0.31	0.28	0.36	<0.001
Diabetes	0.75	0.63	0.88	0.001
Hypertension	0.48	0.42	0.54	<0.001
Kidney diseases	0.92	0.51	1.69	0.799
Heart diseases	0.83	0.59	1.18	0.305
Lung disease	0.84	0.74	0.95	0.006
Duration of symptoms prior to admission	1.07	1.05	1.09	<0.001
Length of hospital stay	1.07	1.05	1.08	<0.001

Note: For age category: age 41-50 years, residence: urban, department: pediatric, hospital: privet hospital were the reference group. OR: Odds Ratio, aOR: adjusted odds ratio, COPD: Chronic obstructive pulmonary disease
